# Audit on antipsychotic prescribing in children and young people with a learning disability under the care of mental health services in Surrey

**DOI:** 10.1192/bjo.2021.229

**Published:** 2021-06-18

**Authors:** Timothy Cherian James, Asifa Zainab, Salvatore Mura, Aaron Vallance, Dola Okusi

**Affiliations:** 1 Surrey and Borders NHS Foundation Trust; 2 Surrey and Borders Partnership NHS Foundation Trust

## Abstract

**Aims:**

To check the extent to which National Institute of Clinical Excellence (NICE) guidelines were being followed in clinical practice with regards to prescribing antipsychotic medication to Child and Adolescent Mental Health Services (CAMHS) patients with a diagnosed learning disability (LD).

**Method:**

A data collection tool (based on a similar Royal College of Psychiatrists [RCPsych] audit) was filled out with retrospective data from patients’ clinical records, then analysed using Microsoft Excel and Microsoft Powerpoint.

The agreed standards were the NICE guidelines.

There were no ethical issues as the data were retrospective and anonymised.

Sample size was 13, comprising 7 males and 6 females.

All service users were less than 18 years of age.

**Result:**

7 out of the 13 patients who were prescribed antipsychotics had a Severe/Profound LD.

Among the 5 patients who had been prescribed antipsychotic medication, 4 were on Risperidone and 2 were on Aripiprazole. The reasons for starting antipsychotic medication were clearly documented for all 5, the most common reasons being overt aggressive behaviour and general agitation/anxiety.

Only 1 patient had antipsychotic medication initiated in the previous 12 months. NICE guidelines had been generally followed for the management of this case, with good documented evidence.

For the other 4 patients, in whom antipsychotic medication was initiated more than 12 months ago, there was a lack of documentation of the subsequent assessment of side effects, extra-pyramidal side effects, body weight, blood pressure, glycaemic control and lipid profile. 1 of these patients did not have a documented review of antipsychotic medication in the previous 6 months. For the other 3 patients, their medication reviews did not consider whether to reduce the dose or stop antipsychotic medication.

1 patient had been transferred to primary care, with a clear transfer of prescribing responsibility and documented evidence that written guidance was provided to primary care which addressed all the necessary management details.

**Conclusion:**

Although there was clear documentation of reasons for initiating antipsychotics, there appeared to be a lack of awareness of NICE guidelines for antipsychotic medication reviews, side effect and metabolic markers assessment, and their documentation. This is an area for potential change in practice to conform better to national guidelines and improve patient care.

